# A Beautiful Compliment to the Physician

**Published:** 1856-01

**Authors:** 


A Beautiful Compliment to the Physician.—I dare not place any gift, however beautiful, or any success however brilliant, above the talent or the skill which can relieve a single pang, and the self-devotion which lays them at the feet of the humblest fellow creature.—*Oliver Wendell Holmes.*

